# Fighting Iron-Deficiency Anemia: Innovations in Food Fortificants and Biofortification Strategies

**DOI:** 10.3390/foods9121871

**Published:** 2020-12-15

**Authors:** Ângela Liberal, José Pinela, Ana Maria Vívar-Quintana, Isabel C. F. R. Ferreira, Lillian Barros

**Affiliations:** 1 Centro de Investigação de Montanha (CIMO), Instituto Politécnico de Bragança, Campus de Santa Apolónia, 5300-253 Bragança, Portugal; angela.liberal@ipb.pt (Â.L.); iferreira@ipb.pt (I.C.F.R.F.); 2 Tecnología de los Alimentos, Escuela Politécnica Superior de Zamora, Universidad de Salamanca, Avenida Requejo 33, 49022 Zamora, Spain; avivar@usal.es

**Keywords:** iron-deficiency anemia, iron bioavailability, food fortification, dietary strategies, biofortification, agronomic/genetic techniques

## Abstract

Iron deficiency remains one of the main nutritional disorders worldwide and low iron intake and/or bioavailability are currently the major causes of anemia. To fight this public health problem, the scientific challenge is to find an iron form with sufficient bioavailability to increase its levels in humans through food fortification. In turn, biofortification appears as a comparatively advantageous and bearable strategy for the delivery of vitamins and other micronutrients for people without access to a healthy and diverse diet. This approach relies on plant breeding, transgenic techniques, or agronomic practices to obtain a final food product with a higher iron content. It is also known that certain food constituents are able to favor or inhibit iron absorption. The management of these compounds can thus successfully improve the absorption of dietary iron and, ultimately, contribute to fight this disorder present all over the world. This review describes the main causes/manifestations of iron-deficiency anemia, forms of disease prevention and treatment, and the importance of a balanced and preventive diet. A special focus was given to innovative food fortification and biofortification procedures used to improve the iron content in staple food crops.

## 1. Introduction

Iron has been recognized since ancient times as an essential element for human health and well-being [1]. In the early 17th century, this element was exploited as a medicine by some communities, including Egyptians, Hindus, Greeks, and Romans, to deal with chlorosis, a disorder usually caused by iron deficiency [2]. For several years, the interest in iron was focused only on its contribution to the genesis of hemoglobin and transport of oxygen. However, in the last decades, the lack of this element has been associated with a nutritional disorder that causes a large number of cases of anemia worldwide in humans. In developed countries, low iron intake and/or bioavailability are the main factors responsible for this disorder, while these causes correspond to only about half of the cases found in emerging countries [3]. In these territories, infections and inflammatory conditions (particularly malaria), blood loss due to parasitic infections and other nutrient insufficiencies (such as lack of vitamin A, riboflavin, folic acid, and vitamin B12), are also significant causes of anemia [4].

Although theoretically simple, the anticipation of dietary iron deficiency seems intricate at the population level, since the outcome of any element is determined by numerous factors, namely its highly regulated absorption, transport, metabolism, and degree of relationship with physiological courses, etc. [5]. Consequently, iron deficiency arises when its levels in the human body are insufficient, mainly due to its scarce intake, excessive turnover, and/or loss of it [6]. Therefore, iron deficiency persists as one of the most prevalent nutritional disorders worldwide, with a larger incidence in growing children, premenopausal and pregnant women, the elderly, and people with chronic diseases [7], which reflect the physiological needs of dietary iron throughout life periods [8].

As long as vitamin and mineral deficiencies remain to be a serious health problem globally, affecting more than two billion people or one in three individuals [9], and mostly in developing countries, the search for solutions that can reduce the harmful effects of these nutritional disorders is of paramount significance [10]. Therefore, in addition to oral and intravenous supplementation and dietary and nutritional education strategies to safely improve the micronutrient status in the human body, fortification and biofortification approaches emerge as relatively profitable and long-standing micronutrient supply strategies for those who, otherwise, may not have access to or cannot afford to have a fully balanced diet [11]. This approach aims to place the micronutrient dense-trait, such as iron, in staple crops and food products, using different procedures and bioengineering techniques. The fortified/biofortified products, when regularly consumed, will improve the iron levels in the organism safely and effectively and, therefore, promote human health through nutritional benefits [12].

## 2. Iron: A Double-Edged Sword?

Iron is a chemical element that, in addition to being widely distributed on Earth, is biologically necessary for all living organisms [13]. In fact, iron is an ubiquitous cation with a key role in hemoglobin, also essential in cytochromes and enzymatic responses, being a key micronutrient in erythropoietic function, oxidative metabolic rate, and cellular immune responses, also spread equally as an active metabolite and in storage pools [6,14]. In the human body, iron is found mainly in complex arrangements linked to proteins (hemoproteins) such as heme compounds (hemoglobin or myoglobin), heme enzymes, or non-heme compounds (flavin-iron enzymes, transferrin, and ferritin). Almost two-thirds of all iron in the human body is established in the hemoglobin of circulating erythrocytes, 25% is confined to an easily transferable iron supply, and the lasting 15% remains connected to myoglobin in the muscle tissue and in a variety of enzymes involved in the oxidative metabolism and many other cellular functions [15]. Therefore, every human cell has an unconditional need for iron [16]. The unique characteristics that make iron valuable for standard cellular functions, likewise make it able to catalyze reactions that result in the origin of reactive oxygen species (ROS), which occurs when iron levels are very high [17]. Therefore, although iron is an essential micronutrient in the human organism, it can also be toxic when present in excessive concentrations. To ensure that iron remains at its optimal physiological level, both individual cells and the entire organism have developed mechanisms to safeguard this level, namely through coordination achieved by two homologous ribonucleic acid (RNA) binding proteins and a liver-derived peptide that plays an important role at the whole body level [18,19].

## 3. Iron Deficiency

Iron deficiency ranges from iron-depleted states without anemia to more severe conditions with extreme hematological manifestations, which correspond to the most clinically severe scenario. However, barring massive blood loss, the evolution from iron deficiency to iron-deficiency anemia is generally slow. It is also known that iron deficiency can be caused simultaneously by several factors. In developing countries, poverty, malnutrition, hunger, hookworm infections, and schistosomiasis, which leads to chronic blood loss [20], are clear causes of anemia among iron-deficient people. On the other hand, the main reasons for iron-deficiency anemia in industrialized countries are related to severe vegan and vegetarian regimes, malabsorption, and chronic blood loss caused by menstrual periods. In older people, the prevalence of anemia is associated with progressive age and various connected disorders, such as inflammation, diminished ranks of erythropoietin, and cancer [21]. Other conditions such as obesity [22], congestive heart failure [23], and genetic causes may be underlying iron-deficiency anemia.

This micronutrient disorder is chronic, commonly asymptomatic and, consequently, frequently undiagnosed. However, there are many data in the literature that point to the adverse outcomes of this condition on children’s cognitive development, physical performance, working productivity in adults, and consequences for pregnancy [8]. Other symptoms, such as pallor, fatigue, dyspnea, headache, and alopecia are common in this type of anemia. It has also been described that this disorder can cause changes in thermogenesis and thyroid metabolism, but it may also increase irritability muscle weakness and susceptibility to infections, etc. [8,23,24,25].

**Figure 1:**
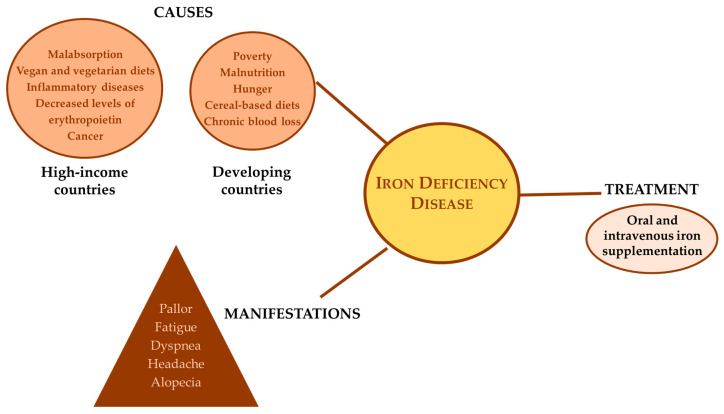
Main causes, manifestations, and current treatments for iron-deficiency in humans.

Given its negative effects, iron-deficiency anemia needs to always be handled by dietary iron supplementation. Generally, the treatment of iron deficiencies comprises two main therapeutic approaches, applied through oral and intravenous formulations, chosen considering the severity of iron deficiency or anemia, the amount of this element that must be replaced, and the presence of an active inflammation, etc. The oral iron supplementation is usually the first line of treatment for this disorder, as it is an appropriate, economical, and effective option for handling with stable patients [26]. However, this approach has potential disadvantages, especially in terms of gastrointestinal adverse reactions such as dyspepsia, nausea, vomiting, abdominal discomfort, constipation, and diarrhea, etc., which limit the patients’ adherence to this specific therapy [27]. Alternatively, the intravenous iron therapy can be considered where the administration route allows the calculation and prescription of the appropriate iron dose required for the normalization of hemoglobin values and recovery of iron reserves, which constitutes an important advantage of this therapeutic option [8]. Figure 1 summarizes the major causes, manifestations, and treatments for iron deficiency.

## 4. Iron Absorption and Regulation Mechanisms

The two main sources of the iron used by the human body came from food and the recycling of senescent erythrocytes [28], through the iron absorption cycle, which is also induced by endogenous and exogenous features, such as individualities of the ingested form of iron and food matrix [29]. In cells, iron is transported to the mitochondria for the synthesis of heme or iron-sulphur clusters, while the extra amount is stored either as ferritin or hemosiderin and purified in cytosolic ferritin [5,6]. Since most of the iron in the human body remains bound to hemoglobin, phagocytosis and abasement of senescent erythrocytes characterize the key foundation of this element (25 to 30 mg/day), whose quantity is sufficient to maintain the daily iron requirements for erythropoiesis [30].

### 4.1. Effect of a Balanced Diet on the Iron Bioavailability and Absorption

It is well established that the bioavailability of iron is as important as the total iron in the diet. In this field, the chemical arrangement of this element plays an important role, since the two existing iron forms (heme and non-heme) are primarily obtained from different sources. The heme form is mainly present in flesh food, such as meat, poultry, and fish, and has more bioavailability than the non-heme form, obtained, in different amounts, mainly from plants and animal foods [29]. The absorption of non-heme forms by the organism must be improved by other ingredients, such as ascorbic acid and flesh food, which can enhance the absorption of this metal, achieved by adopting a balanced diet. Hence, dietary options and choices can substantially provide dietary compounds that either improve or obstruct the absorption of non-heme iron, mainly in plant-based diets, where inhibitory compounds predominate. Depending on the ingested quantity, a balance between enhancers and inhibitors can compensate the inhibitory effect of high intakes of these compounds [5,31].

#### Enhancers and Inhibitors of Iron Absorption

When routinely ingested in a meal, nutritional and antinutritional compounds can considerably improve or inhibit iron absorption, respectively. In fact, decreasing antinutrients intake appears to be the smart approach to improving the iron absorption by the human body [32]. For example, the adoption of a plant-based diet, which is gaining even more popularity due to its role in sustainable, low-meat and healthy diets in high-income countries, is the reason for the low bioavailability of this element, given the non-heme iron (<10%) malabsorption when compared to the heme form (15–35%), mainly found in animal tissues [33]. Specifically, legumes are characteristically rich in proteins, fibers, resistant starch, polyphenols, and a wide range of micronutrients, thus holding a strong potential for combating nutritional disorders [34]. However, in addition to their well-known nutritional quality, legumes include in their composition several antinutritional factors, some of which have been described as inhibitors of iron absorption by the human body, such as some phenolic compounds and phytates [35,36]. Moreover, cereals and fruits hold high amounts of phytic acid, iron-binding phenolic compounds, and calcium, which also potentially obstruct the iron absorption by animals [37]. However, it is well established that traditional food processing methods, such as soaking, malting, and fermentation can thus improve iron absorption from phytate-rich foods, since these approaches are capable of dephosphorylate phytate from the myo-inositol hexaphosphate form into lower myo-inositol phosphates [38]. Other reports also point tannins as iron inhibitors, even in the presence of an iron absorption enhancer or cereal-based fortified foods [32]. Animal proteins such as milk, eggs, soybean proteins, and albumin also seem to hinder iron absorption. Yet, one study [39] reported that adding small amounts of pork meat to a phytate-rich meal can enhance iron absorption by 57%. Literature data suggest that products moderately assimilated from animal tissues have the ability to fix iron through their histidine and cysteine residues, which, on the other hand, increases iron absorption [29]. Still, these mechanisms need further investigation. Other substances are known for their potential to improve iron absorption, such as ascorbate and citrate, for their role as weak chelators that aid in the solubilization of this metal in duodenum, and for their capability to reduce ferric to ferrous iron. Furthermore, ascorbic acid compensates for the harmful effects of all inhibitors, including phytates, calcium, and proteins in milk products [40], increasing the absorption of natural and fortifying iron. On the other hand, in fruits and vegetables, the potentiating outcome of ascorbic acid is offset by the inhibitory influence of polyphenols [41].

In vegetarian diets, the only iron absorption enhancer is ascorbic acid, whose activity can be improved by the consumption of other vegetables that also hold it. The enhancer effect of ascorbic acid was reported in a study where vitamin C from maize and wheat improves iron absorption up to 84% and 48%, respectively, in these grains [42]. Other well-known enhancers of iron absorption by the organism are folic and citric acids, peptides rich in the amino acid cysteine, and vitamin A [32]. Other interactions between iron and additional trace elements such as manganese (Mn), zinc (Zn), and chromium (Cr), also appear to have an influence in iron metabolism and absorption [43]. Previous research shows that the similarity in the transport and absorption mechanisms of iron and Mn are the basis of their interaction [44]. In humans, this association occurs mainly in iron-deficiency states, where an extreme Mn intake can result in exacerbated iron-deficiency and Mn toxicity [45]. Regardless of the interplay between iron and Zn, especially in the presence of iron-deficiency and Fe supplementation, the data are contradictory, since a number of factors can dictate whether Zn increases or decreases iron absorption, and vice versa [43]. In turn, Cr × iron interaction in the human organism appears to be affected by several factors (pre-existing diseases, nutritional status, and diet). However, the specific characteristics of such relations and its basic mechanisms are, until recently, not well understood [43].

### 4.2. Recommended Dietary Allowances (RDA) of Iron

Current iron RDA for non-vegetarians are listed in Table 1. For vegetarians, RDAs are 1.8 times greater than for those who consume meat products, as heme iron from meat products is more bioavailable than non-heme iron from plant-based foods. For children from birth to 6 months, the Food and Nutritional board established an adequate intake (AI) of iron which is comparable to the average iron intake in breastfed healthy children. Iron requirements also increase 3-fold during pregnancy due to an enlargement of the maternal red-cell mass and fetal-placental progression. Subsequently, during postnatal development, the human body still has very high iron needs, which can be achieved through the dietary intake of this essential element. However, iron requirements decrease and levels in the human body remain relatively constant once adulthood is reached. Each day, the newly absorbed iron supplants the small quantities of the lost iron (1–2 mg) [46]. Accidentally poisoning with medicinal iron has been reported in which an oral dosage of 60 mg/kg body weight may be fatal. Typically, poisoned individuals express symptoms such as nausea, vomiting, and lethargy or coma, then an asymptomatic period for up to 24 h, which is followed by gastrointestinal perforation, convulsions, cardiovascular failure, and hepatic and renal collapse [15]. The tolerable upper intake level (UL) of iron for adults is 45 mg/day, a level manifested by gastrointestinal distress as a hostile outcome [15].

## 5. Dietary Strategies for Addressing Iron Deficiencies

Food products are one of the main recognized dietary bases of iron, and its bioavailability, as mentioned above, is extremely dependent on its chemical arrangement, nutritional features, and concentration. In the last few years, the growing number of people affected by the lack of this micronutrient has promoted the development of non-therapeutic techniques that aim to suppress the iron deficiency in the human organism. In addition to the consumption of iron-rich foods, (bio)fortification approaches give rise to a variety of techniques that aim to compensate the lack of this element and its side effects, increasing its bioavailability to be absorbed by the human organism [15].

### 5.1. Innovative Food Fortification and Biofortification Procedures

#### 5.1.1. Fortification Approaches

Iron fortification is the practice of providing an additional amount of iron to food matrices to increase their nutritive value and their ability to provide public health benefits [47]. Despite being a simple, inexpensive, and feasible process, iron is the hardest micronutrient to generate fortified foods, since it is relatively high reactive and oxidizable depending on temperature and air contact, which can result in undesirable organoleptic fluctuations [48]. The oxidation process can modify other food constituents and alter the color of food vehicles, problems that may reduce their quality, promote hostile sensory characteristics, and reduce the shelf-life of the final product [49]. Therefore, the scientific challenge is to achieve an iron form that can afford enough bioavailability to improve iron assimilation, when added in adequate amounts, and which is not discarded due to sensory deviations or hostile gastrointestinal outcomes [50]. The iron content in some iron-fortified crops compared to their normal values, are shown in Table 1.

**Table 1:** Iron concentration in normal and fortified crops [51].

Plant Food	Typical Iron Content (µg/g) ^a^	Biofortification Target (mg/g dw)	Fold Increase	Reference
Rice, brown	15			[52]
Rice, polished	2	15	7.5×	[53]
Wheat, whole meal	30	59	2×	[52]
Wheat, flour, white	7			[54]
Maize, whole	30	60	2×	[52]
Common bean	50	107	2.1×	[53]
Peas, dried	50			[53]
Pearl millet	47	88	1.9×	[55]
Cassava root	5	45	9×	[53]
Sweet potato	6	85	14.2×	[56]
Irish potato	3			[57]
Cabbage, broccoli	17			
Tomatoes	5			
Beef steak	35			

^a^ Results expressed in µg/g of plant food, in “fresh weight” for cassava, potatoes, and vegetables.

Regardless of the form of iron administration, the target population is one of the main criteria to be considered. Therefore, food fortification provides an easier interaction with the daily nutritional needs of rural communities, where access to food supplements is less common [32]. Although the effects of this approach are not as fast as those of oral and intravenous supplementation, this represents a useful method to deliver iron through foods that follow the normal absorption mechanisms of a daily diet, leading to a gradual increase in the population’s iron levels, along with sustainable long-term benefits [58].

The success of the iron fortification approach depends on three main characteristics, including the vehicle selected for its inclusion, the iron compound, and its baseline nutritional deficiency prevalence. Although numerous fortifiers are accessible for this purpose, the challenge is to find an iron compound that offers better absorption by the human organism and, at the same time, that does not modify the individual characteristics of the food vehicle. Some aspects such as solubility, feasibility of application, and wanted bioavailability should be considered, and preference should be given to a compound that has less bioavailability but can be supplied in larger quantities without adverse organoleptic outcomes [32].

Meanwhile, the study of new iron fortificants is in vogue and the European Food Safety Authority (EFSA) has established that: Ferrous bisglycinate; ferric sodium EDTA (or sodium ferric ethylenediaminetetraacetate, whose exposure to EDTA should not exceed 1.9 mg EDTA/kg body weight/day) [59]; iron (II) taurate [60]; and iron L-pidolate [61] are safe iron sources for fortifying purposes. Additionally, the recommendations of the World Health Organization (WHO) point out ferrous sulfate, ferrous fumarate, ferric pyrophosphate, and electrolytic iron as preferred compounds for the cereals fortification with iron [62]. The appropriate combination of vehicle and fortifier to be used in the development of iron-rich foods is of high importance. Once again, the target group that will benefit from its intake should also be taken into account. Therefore, the vehicle must be a foodstuff regularly consumed by this population group to achieve high micronutrients bioavailability [33]. Recent studies of iron fortification approaches are described in Table 2. Good examples of this feature are the infant formula, as they hold ferrous sulfate, which is 100% soluble when the formulation is reconstructed, and ascorbic acid, which assurances enough iron bioavailability [63,64]. In a study performed by Shama-Levy et al. [63] to verify the efficiency of adding ferrous sulfate and ferrous gluconate as fortifiers in infant formulas, children from 12 to 30 months of age were randomly assigned to receive three versions of the initial formula: One fortified with ferrous sulfate, another with ferrous gluconate, and the last with no fortifiers, for a period of 6 months, with hemoglobin, serum ferritin, and soluble transferrin (sTfR) receptors evaluated in the final blood samples. The results showed that the fortification of the infant formula with ferrous gluconate had a beneficial effect on the iron status markers. This proven efficacy, together with other sensory data, led to the political decision to replace iron with hydrogen reduction, formerly used as a fortifier, with ferrous gluconate. The food matrix composition must also be taken into account, since the effects of enhancers and inhibitors are crucial for the success of the fortification process [65]. In a previous study [66], the effect of ingesting a fortified fruit juice with microencapsulated iron pyrophosphate coated with lecithin on the iron status of menstruating women was investigated. In this 16-week double-blind, placebo-controlled study, individuals were randomly divided into two groups, which consumed, in addition to the usual diet, 500 mL/day of fruit juice with placebo or iron-fortified, in which the last provided 18 mg iron/day (100% RDA). Food intake, body weight, and varied parameters of iron were assessed at the beginning of the study and monthly. The results showed that the iron-fortified juice increased the iron levels, showing that this iron form in this specific food matrix is effective in increasing the bioavailability of this element in iron-deficient women. However, the same form of iron, when administered to a similar group of women with an iron deficiency, was not as effective when added to a dairy product, in a study performed based on the same methodology. This can be explained by the presence of different compounds in the two food products, since the fruit juice is rich in ascorbic acid and the last in calcium, which are potentiators and inhibitors of iron absorption, respectively [67]. These findings support the food vehicle’s reputation, since, although encapsulation can prevent iron from interacting with other food constituents, experimental results with animal models and humans showed that, no matter how good the fortifier is, its consumption in combination with iron enhancers or inhibitors regulates its final outcome [68]. Other foods such as cereal-based products, dairy products, seasonings (salt, soy sauce, fish sauce, bouillon, curry powder, sugar, etc.), and legumes, such as lentils [69,70], are already used for this purpose, since they are staple foods consumed worldwide [71]. For example, white rice is a cereal that contains low iron levels and is essentially predominant as a staple food in regions with a higher incidence of anemia [72]. Typical rice fortification approaches include dusting, coating, steaming, and extrusion. Nevertheless, some weaknesses of these methods have been reported, mainly with regard to impacts on nutritional value and bioavailability. Given the disadvantages of the mentioned approaches, potential applications of cold plasma processing in cereals and pulses have been investigated in recent years, since this treatment preserves nutritional and textural attributes with a reduced processing time [73]. Once cold plasma generates an acidic environment and given its surface etching and cross-linking assets, it can benefit the absorption and linkage of iron on the rice surface, while reducing iron oxidation during storage. In this sense, an unprecedented investigation was carried out on the role of cold plasma processing in the fortification of rice with iron and on the bioavailability of this element through storage [74]. For the effect, rice samples were processed with plasma at a constant voltage of 20 kV, for 10 and 15 min, based on surface characteristics, hydrophilicity, and thermal assets. An increased bioavailability of free ferrous iron state (Fe^2+^) in the fortified rice after the plasma treatment compared to the control group, as well as a prolonged storage time without changing the rice characteristics were observed. Regarding seasonings, Karn et al. [75] performed a study in 40 Nepalese households and 10 restaurants to evaluate the bioavailability of different iron fortificants in curry powder and their effects in its qualities. For that, sources with good bioavailability of iron, such as ferrous sulfate, ferrous fumarate, and sodium ferric ethylenediaminetetraacetic acid (NaFeEDTA), were added to provide one-third of the recommended daily intake (RDI) of iron per serving. The results show no adverse effects on the physical, chemical, and sensory qualities of curry powder and that relative bioavailabilities of NaFeEDTA were 1.05 times higher than that of ferrous sulfate, concluding that it is feasible to fortify Nepalese curry powder to address iron-deficiencies.

As research on new iron fortifiers is under development, nanotechnology engineering has emerged to provide iron forms and vehicles that can be highly absorbed by physiological pathways [76]. Dairy products are part of the group of foods in which iron fortification is difficult, as certain characteristics such as color, taste, odor, and texture can be modified due to the oxidation of the free ferrous iron state (Fe^2+^) into the ferric state (Fe^3+^) [77], and the metallic taste of iron salts may limit its intake. This has driven the search of a novel approach of fortification for these products. Therefore, Cheng et al. [78] developed ferrous sulfate liposomes as an iron delivery system that aims to fortify dairy products such as milk, which proved to be highly effective in the encapsulation process, showed good oxidative stability and biocompatibility, and low cytotoxicity when compared to other methods, therefore being considered an efficient fortification method. In this field, liposomal microencapsulation presents a technology that protects iron from the straight interaction with other components of the food vehicle, resulting in minimal oxidation, improved iron bioavailability, and compensation for sensory changes [79]. Overall, the results achieved by Cheng et al. [78] evidenced that ferrous sulfate liposomes have high encapsulation capacity, good oxidative stability and biocompatibility, and low cytotoxicity, with improved hemoglobin concentration in iron-deficient rats, making this approach suitable to iron fortification. Other techniques that aim to reduce the iron particle size are also under investigation. Cakmak et al. [80] noticed that in an animal model, the Fe (III) oxide nanoparticles were absorbed by the ferric route and that did not cause hostile hematological outcomes, suggesting that it is an effective approach that gives rise to iron arrangements and fractions that controls iron solubility and absorption as desired.

Additionally, ferritin nanoparticles can be good iron suppliers, since ferritin is a large protein enabled to store large amounts of iron. However, its absorption mechanism is not yet well known [78,81]. Encapsulation is a technique that allows the controlled and targeted release of a certain component, trapped in a surrounding matrix. Generally, the encapsulated, lipid-based forms of iron complexes are indicated for food ingredients and products, such as baby foods, dry mixtures for drinks, and poorly processed foods, and help prevent color deterioration and improve the bioavailability of the carried element. Ferrous sulfate and encapsulated ferrous fumarate are used as fortifiers for infant formulas and wheat and corn cereal flours [50].

**Table 2:** Examples of recent studies of food fortification with iron.

Fortification Approach	Food Vehicle	Key Outcomes	Reference
Ferrous sulfate and ferrous gluconate enhancement	Infant formula	Both ferrous sulfate and ferrous gluconate were successful when added as fortifiers in an infant formula, which showed positive effects on the iron status markers, namely in ferritin and sTfR levels and in body iron stores.	[63]
Microencapsulated iron pyrophosphate	Fruit juice	The daily consumption of a microencapsulated iron pyrophosphate-fortified fruit juice shows an increase in Fe status in a short period of time (4 weeks). This consumption was well-suited with the traditional diet, and the extra daily 18 mg of Fe provided was 100% of the RDA.	[66]
	Dairy products	Iron-fortified seasoned skim milk does not expand the iron status in iron-deficient menstruating women due to the presence of calcium, a well-known iron absorption inhibitor.	[67]
Treatment with cold plasma	Rice	Improved bioavailability of the free ferrous iron state (Fe^2+^) in the fortified rice next to cold plasma processing. Extended storage time without changing the rice characteristics.	[74]
Ferrous sulfate, ferrous fumarate, and NaFeEDTA enhancement	Curry powder	No hostile effects on the physical, chemical, and sensory attributes of curry powder. Bioavailability of NaFeEDTA were 1.05 times higher than that of ferrous sulfate.	[75]
Microencapsulation of ferrous sulfate	Liposomes	The approach applied to dairy products proved to be extremely effective, showing good oxidative stability and biocompatibility, and low cytotoxicity, once the encapsulation protects iron from the straight interaction with other components of the food vehicle.	[78]
Ferritin enhancement	Nanoparticles	Good iron supplier, since ferritin holds large amounts of iron.	[81]

#### 5.1.2. Biofortification Procedures

Since most food products consumed by humans come from plants, a relationship can be established between agriculture and nourishment to promote human health and well-being. In this field, biofortification emerges as a technique that aims to enhance plant crops with vital macro- and micronutrients through biotechnology processes [82]. The basic objective of this technique is to decrease the mortality and morbidity rates associated to micronutrient malnutrition and gives rise to food security, efficiency, and quality of life for deprived populations in emerging countries [12]. For that, certain parameters must be established, including the safety of the final product, the cost-benefit, and the low environmental impact resulting from the process implementation [83]. There are three classic approaches to the biofortification of stable crops, namely conventional, agronomic, and transgenic biofortification, each focused on different parameters, but with the same final purpose.

##### Conventional Biofortification

Conventional biofortification of plants has been carried out for centuries through the crossing of species with the desired characteristics, followed by the selection of descendants that hold the agronomic characteristics inherited from both parents, with the main objective of improving the characteristics of food crops [84]. Cultures biofortified using this method require a high degree of genetic variability in the populations used for the chosen features, such as a high content of essential elements such as iron, zinc, etc. As breeding strategies often depend on this variability, in some cases these species may be crossed with distant relatives, allowing a slow transfer of the desired characteristics to commercial cultivars. In addition, other new features can be directly presented in these descendants through mutagenesis [85]. The biofortification technique by means of genetic breeding is manly used in developing countries, since the population has greater nutritional deficits and has better acceptance of cereal crops. Over the last few decades, genomic advances have evolved to achieve cereal crops that maintain a high-bioavailable element concentration in edible parts [86]. The disparity in the availability of zinc and iron in grains in the target gene pool is a requirement in this field [87]. In certain cereals, such as corn, rice, wheat, barley, and sorghum, there is a significant variation in the amounts of these elements. For example, several studies report that this variability has made it possible to improve wheat cultivars, crossed with closely related wild species [88,89,90,91].

##### Agronomic Biofortification

Agronomic biofortification, traditionally used in the enrichment of grain crops with essential micronutrients, involves the absorption of these elements from the adjacent soil and their translocation to edible portions of the plants through a natural course dependent on the bioavailability of the micronutrients in question. The effectiveness of this approach depends on several factors, as there is a potential loss of nutrients during these transition stages [86]. The management of the soil to enhance its properties include some strategies such as the use of organic wastes, such as plants crop remains, animal manure, and other compounds, not only to expand its assets, but also the water retention capacity and the bioavailability of the nutrients, released gradually and slowly, etc. [92].

In plants, several nutrients are known to improve the intake and remobilization of certain elements, such as iron, developing specific strategies for the effect [93]. Some of these strategies include an increased exudation of phytosiderophores (PS), which form stable complexes with Fe^3+^ subsequently absorbed by the roots, by some graminaceous species [94]. The sulfur-containing amino acid methionine is the only precursor of PS and, given this assumption, the iron metabolism in plants is closely related to sulfur, mainly in cereals [95]. Astolfi et al. [96] completed a study focused on the possible outcomes of sulfur application rates and concentration on durum wheat plants competence to gather iron in grains under iron deficient conditions. Their results revealed that a super optimal sulfur fertilization helped the plants gather higher amounts of iron in sprouts, mitigating the outcomes of iron deficiency. On the other hand, the same authors report that, a high sulfur supply did not improve the iron concentration in the grains, showing that this concentration was markedly higher in the seeds formed by plants that grew with lower amounts of sulfate, which suggests that the mechanisms that regulate the allocation of iron in seeds might be different from those that regulate the root acceptance and allocation in the leaves of this micronutrient. Another study also reported that the combination of a nitrogen fertilizer with iron applied in soil or leaves improves the yield and uptake of this element [97].

In contrast, the continuous application of fertilizers over prolonged periods of time can lead to the possible accumulation of toxic compounds in the soil, leading to their ecological deterioration by heavy metals, nitrates, and other substances potentially harmful to food crops and human health [98], which can lead to the higher incidence of several types of cancer [99]. In this line, nanochelating technologies emerge as a novel approach applied to the synthesis of nanostructures capable of reducing the harmful impact of conventional fertilizers. Fakharzadeh et al. [100] developed a nanochelated iron fertilizer, used in several development periods, to evaluate the response of rice productivity, some agronomic traits, grain protein content, and nutrients concentration after application of the nanochelated iron fertilizer. These results showed that the foliar application of nanochelated iron fertilizer in paddy fields offers an efficient strategy for the biofortification of rice, since the nanochelated fertilizer enhanced plant stature, panicle size, grain weight, and paddy yield and, additionally, supplemented the white rice with nitrogen, phosphorus, and potassium as compared to the control. Moreover, the foliar application of 2.5 g/L of nanochelated iron fertilizer at nursery and booting points had extreme results in the rice quality and quantity parameters, with a cost-effective average and with a low application of the nanochelated iron fertilizer.

##### Genetic Biofortification

Genetic engineering approaches, when compared to conventional and agronomic plant breeding practices, are additionally effective and consistent to study the genotypic and phenotypic associations between plants [101]. The main objective of genetic biofortification is to improve the bioavailability of micronutrients in edible parts of plants [102], currently assuming itself as the preferred approach applied to grains. Genetic biofortification is based on access to a limitless gene pool for the allocation and expression of the required genes from one plant species to another, regardless of their evolutionary state or taxonomy, in order to manipulate the organism’s nucleic acid. The result of this technique is recognized as a genetically modified organism (GMO) [86].

Important enzymes such as nicotinamide synthase, phytoene synthase (PSY), ferritin, and carotene desaturase have been used in various crops to add or improve certain nutritional properties. As nicotinamide is a metal chelator that acts as one of the main constituents in the integration and homeostasis of metals, the manipulation of its concentration can improve the iron amounts in plants [103]. Moreover, ferritin, the key iron storage proteins in all existing aerobic organisms, can promote the iron storage capacity of the grain endosperm over its overexpression, thereby improving the iron content in plant foods [104]. Moreover, when a certain micronutrient is not naturally present in crops, transgenic procedures are the only feasible possibility of fortifying these products with specific nutrients [105]. Genome editing methods, such as the introduction of genes with the desired characteristics from other organisms, overexpression of these same genes, gene silencing interference (RNAi), and gene knockout, are transgenic approaches used to describe the purpose of genes as substitutes when there is insufficient genetic dissimilarity in the amounts of nutrients between plant varieties [101]. In this field, transcription activator-like effector nucleases (TALENs) and clustered regularly interspaced short palindromic repeats (CRISPR) and CRISPR-associated protein 9 (Cas9) are systems used for crop biofortification, capable of promoting a well succeeded gene-editing without disturbing the entire organism [106]. Genetic biofortification by means of transcription factors has less strict guidelines and monitoring of both governmental and non-governmental figures when compared to GMO crops, then holding greater acceptance by the consumer. This knowledge has benefited from the speed and low cost of next generation sequencing approaches that simplify the non-targeted gene and allele detection [102]. Furthermore, it is a current exercise to associate different molecular breeding and genomic procedures, such as marker-assisted selection, association genetics, and high-throughput phenotyping and genotyping for specific traits [107]. Transgenic methods can be used for the simultaneous integration of genes tangled in the improvement of the micronutrient bioavailability (decreasing the inhibitors and improving the iron absorption enhancers mentioned above) and their concentration in plants [86]. A previous study reported that the expression of the TOM1 gene is related to the outflow of deoxymugineic acid (DMA), a primary member of the PS family. The release of DMA has been described as being improved under iron-deficiency circumstances in diploid, tetraploid, and hexaploid wheat, suggesting a significant role for DMA in iron acceptance in wheat [108].

Through the introduction of metagenomics and next-generation sequencing tools and the expansion of the “holobiome” notion, the significance of the microbiome in the soil and the crop efficiency are becoming more obvious [109]. The main drivers for maintaining soil well-being and crop efficiency are the links between the aboveground and belowground biota of plants and microorganisms, which are recognized for having coevolved in nature [110,111]. The rhizosphere is a metabolically dynamic site where microorganisms sequester, assemble, and make suitable both macro- and micronutrients for plants, and the rates propose that nearly about 20,000 plant species revealed require symbiotic relations with microorganisms for their subsistence and proliferation [112]. Therefore, the crop biofortification approach can be accomplished over the presentation of microbial inoculants/biofertilizers, which organize, solubilize the critical mineral micronutrients in the soil, and make it straightforwardly accessible to the plant. Among the different groups of microorganisms, plant growth-promoting rhizobacteria (PGPR) are recognized for employing one or more direct and secondary mechanisms of action to increase plant development and well-being, even though the main approach for action is to improve the accessibility of nutrients to the plant in the rhizosphere area [113,114]. Micronutrients exists in soils as free or adsorbed ions onto mineral or organic surfaces, or as precipitates within the soil biota. The uptake of these micronutrients from the rhizosphere is the main phase during the build-up in the plant, where PGPR plays an important role in its solubilization and in improving its availability to the plants. PGPR produces plant growth-promoting compounds and mineral solubilizing enzymes and plays a central role in the cycling of macro- and micronutrients, by adjusting the core morphology, causing a larger root surface area for the uptake of nutrients in the soil. The acquisition of mineral elements with limited movement in the soil, such as iron, phosphor, potassium, zinc, and copper, can be enhanced by the emergence of a more general root structure, promoted with the application of biofertilizers in the wheat crop [113,114].

### 5.2. Risks and Benefits of the Different Iron Supplementation Approaches: Balancing Efficacy and Safety

Several strategies are known and implemented to reduce and/or treat iron deficiency and the related anemia at the population level or targeting specific age/consumer groups. Among them are the modification of dietary patterns, which aim to increase the amount and bioavailability of iron to be used by the human organism [115], and different strategies for fortification and biofortification of staple foods/crops, respectively [116].

The iron fortification strategy for food products is subject to the addition of either isolated iron compounds (e.g., iron salts or chelates) or food sources rich in this element (e.g., meat and meat products). To ensure significant benefits to the consumer, the minimum amounts of micronutrients that must be achieved have been defined by regulatory bodies and health authorities. In this sense, the WHO and the *Codex Alimentarius* of the Food and Agricultural Organization of the United Nations (FAO) [117] specify that, for a product to be considered “iron fortified”, it must contain at least 15% of the nutrient reference value (NRV). In turn, it must contain 30% of the NRV to be claimed “rich in iron”. The effectiveness of a particular iron fortification strategy is considered when its status, in women and children fed with products enriched with this element, is maintained or improved for at least 6 months and when carried out in accordance with international recommendations [118]. An increased risk associated with iron interventions occurs in developing countries, where widespread illnesses and a lack of healthcare still predominate. However, effects that promote the intensity of iron-deficiency anemia, as well as adverse effects such as diarrhea due to iron fortification of staple foods, condiments, and infant foods, have never been reported, thus constituting a safer approach to iron administration in low- and middle-income countries. On the other hand, with regard to developed countries, the iron supply is increased by the consumption of products fortified with this element, with the concern that high iron stocks in the human body may be critical factors in cardiovascular diseases, type 2 diabetes, and cancer, a hypothesis supported by the fact that serum ferritin is used as a marker of iron stocks. However, this is an acute phase protein, not considered a good biomarker of the level of iron in inflammatory cases, common in chronic diseases, so there is limited support between its relationship. In short, the reduced risk of adverse factors resulting from iron fortification, both in developed and developing countries, is largely outweighed by the benefits they provide [118]. Furthermore, Eichler et al. [119] argue that references for iron supplements may not be useful for fortified foods, since the regular amounts of the micronutrient provided by this approach are much lower than those achieved by oral and intravenous supplementation. In this sense, the iron fortification of food matrices with recommended iron doses equally diluted in a higher food weight remains one of the harmless approaches accessible to reduce the risk for this micronutrient deficiency.

At the same time, the biofortification of staple crops, compared to other methods, is a more effective and less costly, more sustainable, and long-term means of providing a greater number of essential micronutrients. Actually, no single intervention, including biofortification, is able to fully supply or treat deficiencies in certain micronutrients in all population groups, presenting itself only as a complement to existing interventions for this purpose, and which constitutes as a more sustainable, cheap, and economical way to achieve the same goal [120]. This is particularly more feasible in malnourished rural populations, with difficult access to certain types of diet and fortified products, but it can also reach consumers in urban areas, unlike other interventions that aim at the same objectives. Regarding the costs related to the biofortification of staple crops, apart from the continuous and necessary financial expenses for commercial fortification programs, an isolated investment in the cultivation of plants can yield sufficient micronutrient-rich planting materials for their cultivation for one year [121]. Despite these current expenses, their amount is lower when compared to the cost of the initial development of nutritionally improved crops and the institutional establishment of their nutritional content.

## 6. Conclusions and Future Trends

Eradication of iron “hidden hunger” is the main research objective of the scientific community. Biofortification appears as a realistic strategy for undernourished populations in quite isolated rural regions, carrying naturally fortified food products to those who have restricted access to commercially advertised products, which are more freely accessible in metropolitan areas. Within the strategy of iron-fortified foods, it is imperative to define the key aspects involved. The iron assortment to be applied as a fortificant and the revolution in the research for new products are the main built-up characteristics. Moreover, in biofortified crops, even in those with the utmost iron enrichment defined so far, the iron amount has been slightly higher than the RDA and lower than that in pharmacological formulations. Therefore, future challenges are linked to the development of crops with higher iron contents and bioavailability for human consumption.

Additional studies that stimulate the search for biofortification strategies capable of reducing the concentration of certain antinutritional factors responsible for lower iron absorption should be carried out. Therefore, the provision of detailed data on the mechanisms that regulate the compartmentalization of iron in the different plant organs is also essential to achieve this goal. Additionally, crops biofortified with prebiotics partially avoid the “iron paradox” triggered by the host-pathogen’s opposition to iron, improving intestinal health, and the immune defense associated with the intestine. Other initiatives aimed at promoting large-scale iron biofortification strategies may alleviate the tendency of certain population groups to develop iron deficiency anemia and contribute to the improvement of overall public health.

In the near future, the combination of the best approaches to fortification, biofortification, and dietary standards should be considered for each country and target population, and better coordination between the different associated programs should be promoted, with the ultimate goal of eradicating or reducing the prevalence of iron-deficiency anemia, especially in developing countries.
